# Beneficial Effect of Heat-Killed Lactic Acid Bacterium *Lactobacillus johnsonii* No. 1088 on Temporal Gastroesophageal Reflux-Related Symptoms in Healthy Volunteers: A Randomized, Placebo-Controlled, Double-Blind, Parallel-Group Study

**DOI:** 10.3390/nu16081230

**Published:** 2024-04-20

**Authors:** Yasuhiko Komatsu, Hiroyasu Miura, Yoshitaka Iwama, Yoshihisa Urita

**Affiliations:** 1 Snowden Co., Ltd., Chiyoda-ku, Tokyo 101-0032, Japan; ya_miura@snowden.co.jp; 2 Nihonbashi Cardiology Clinic, Chuo-ku, Tokyo 103-0001, Japan; yiwama@well-sleep.jp; 3 Department of General Medicine and Emergency Care, Toho University School of Medicine, Ota-ku, Tokyo 143-8541, Japan; foo@med.toho-u.ac.jp

**Keywords:** lactic acid bacteria, gastroesophageal reflux, healthy adult, clinical study, postbiotics, *Lactobacillus johnsoni*

## Abstract

A randomized, placebo-controlled, double-blind, parallel-group clinical study was conducted to examine the effects of ingesting a heat-killed lactic acid bacterium, *Lactobacillus johnsonii* No. 1088 (LJ88) on temporal gastroesophageal reflux-related symptoms in healthy volunteers. A total of 120 healthy Japanese volunteers of both sexes, aged between 21 and 63 years, whose Frequency Scale for the Symptoms of Gastroesophageal Reflux Disease (FSSG) total score was 8 or greater, but who were not diagnosed with functional dyspepsia according to the Rome IV classification, were enrolled. They were randomly assigned to either the LJ88 or placebo group and instructed to ingest the test food (1 billion heat-killed LJ88 or placebo) once a day for six weeks. Gastroesophageal reflux-related symptoms were evaluated using FSSG scores as a primary endpoint. The Gastrointestinal Symptoms Rating Scale (GSRS), stomach state questionnaire, and serum gastrin concentration were used as secondary endpoints. In the FSSG evaluation, the heartburn score was significantly improved at 6 weeks in the LJ88 group compared to the placebo group. No severe adverse events related to the test food were observed. In conclusion, daily ingestion of heat-killed LJ88 improved temporal heartburn symptoms in non-diseased individuals.

## 1. Introduction

In the stressful modern world, the health of the stomach is an important issue for every person to have satisfactory everyday life coping with such stresses. Heartburn is a common symptom related to stress [1,2,3]. Although the standard therapy for gastroesophageal reflux disease (GERD), including symptoms of heartburn, is antacid drugs, including proton pump inhibitors (PPIs) [4,5,6], people who suffer from only mild and temporal heartburn might prefer appropriate functional foods to medical treatments.

Some lactic acid bacterial and bifidobacterial strains exert beneficial effects on the stomach. *Bifidobacterium bifidum* YIT10347 is a probiotic bifidobacterial strain that confers beneficial effects on gastric symptoms, including gastrointestinal discomfort and symptoms such as postprandial discomfort and epigastric pain in healthy adults [7]. *Lactobacillus gasseri* OLL2716 is a probiotic lactic acid bacterial strain reported to have a variety of beneficial effects on the stomach, including functional dyspepsia in *Helicobacter pylori*-infected [8] and uninfected [9] subjects, and subjective evaluation of gastric symptoms in non-diseased subjects [10,11]. Fermented milk containing *Lactobacillus johnsonii* NCC533 has a favorable effect on *Helicobacter pylori*-associated gastritis [12]. Although these bacteria are good for the stomach, all such effects were associated with their properties as probiotics, i.e., “live microorganisms, which when consumed in adequate amounts, confer a health effect on the host” [13].

*Lactobacillus johnsonii* No. 1088 (LJ88) is a lactic acid bacterium isolated from the gastric juice of a healthy Japanese adult [14]. LJ88, as a live bacterium, is highly resistant to acids and exhibits anti-*Helicobacter pylori* activity both in vitro and in vivo [14]. Live LJ88 lowers gastric acidity in a germ-free mouse model, and the underlying mechanism has been proposed to be a decrease in gastrin production [14]. Live LJ88 also inhibits the increase in the number of gastrin-positive cells in the stomach induced by PPI administration in a germ-free mouse model [14]. These results suggest that LJ88 is beneficial to the stomach, similar to the probiotic strains described above. However, unlike those probiotic strains, LJ88 is also beneficial to the stomach in its non-living form, which can be categorized as recently defined postbiotics, i.e., “preparation of inanimate microorganisms and/or their components that confers a health benefit on the host” [15]. Heat-killed LJ88 elicited anti-*Helicobacter pylori* activity both in vitro and in vivo [16], inhibited the increase in the number of gastrin-positive cells in the stomach induced by anti-*Helicobacter pylori* triple therapy including PPI in germ-free mice [17], and improved GERD-related symptoms in healthy adults in a pilot clinical study [18]. For producing and using products containing lactic acid bacteria with health benefits, heat-killed bacteria (postbiotics) are much more useful than live ones (probiotics). This is because the shelf life is longer, and the storage condition is not stricter for heat-killed bacteria than live ones. Therefore, it is important to elucidate health benefits of postbiotic bacteria.

In this study, we conducted a randomized, placebo-controlled, double-blind, parallel-group clinical study to better understand the effects of ingestion of heat-killed LJ88 on temporal gastroesophageal reflux-related symptoms in healthy volunteers, and demonstrated that heat-killed LJ88 improved heartburn symptoms.

## 2. Materials and Methods

### 2.1. Study Design

This randomized, placebo-controlled, double-blind, parallel-group clinical study was conducted in an outpatient setting in Japan between September 2022 and October 2023 (UMIN Clinical Trial Registry number: UMIN000048875). The study protocol was approved by the ethics committee of the Nihonbashi Cardiology Clinic (Tokyo, Japan). Written informed consent was obtained from all participants before enrollment. This study was conducted in accordance with the Declaration of Helsinki: Ethical Principles for Medical Research Involving Human Subjects (adopted by the 64th WMA General Assembly, Fortaleza, Brazil, October 2013) and the Ethical Guidelines for Medical and Health Research Involving Human Subjects in Japan.

**Figure 1:**
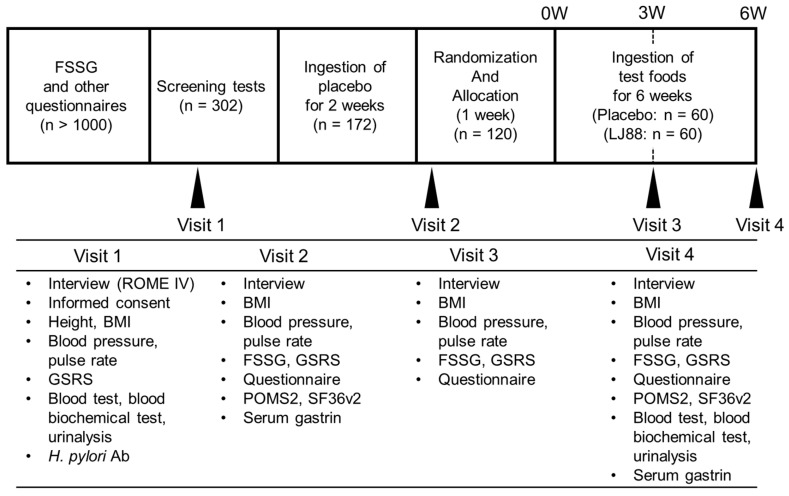
Time schedule of the study and the test items at each visit. Abbreviations used: ROME IV, ROME IV diagnostic criteria for functional dyspepsia; BMI, body mass index; GSRS, Gastrointestinal Symptoms Rating Scale; Ab, antibody; FSSG, Frequency Scale for the Symptoms of Gastroesophageal Reflux Disease; POMS2, Profile of Mood States, second edition; SF36v2, 36-item Short-Form Health Survey v2.

**Figure 2:**
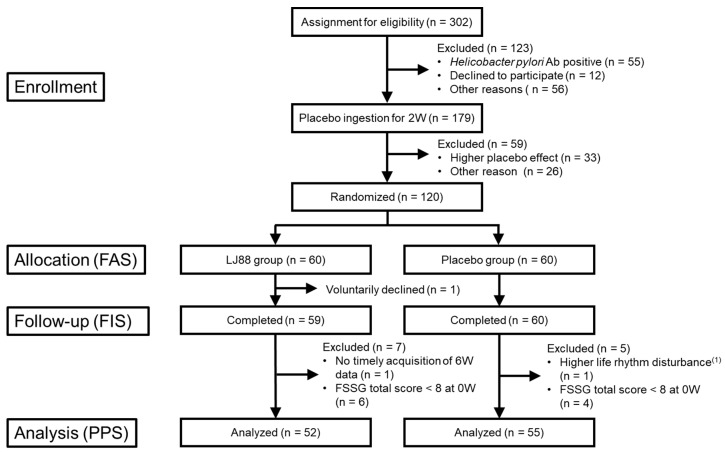
Flow diagram of the study. Abbreviations used: FSSG, Frequency Scale for the Symptoms of Gastroesophageal Reflux Disease; FAS, full analysis set; FIS, full intake set; PPS, per-protocol set. ^(1)^ Moving house during the test period.

The ingestion of the test foods was divided into two periods (Figure 1). The first period started after the screening tests, in which all participants were instructed to ingest the placebo food once a day for two weeks. By using this ‘placebo-ingestion period’, we selected more appropriate participants (n = 120) with lower placebo effect and higher compliance to the instructions. The selected 120 participants were divided into placebo or LJ88 groups and instructed to ingest either placebo food or test food containing heat-killed LJ88 (1 billion cells/day), daily, for 6 weeks. The test items at each visit are summarized in Figure 1. Randomization was performed by a controller (Tsurumi University, Kanagawa, Japan) with a stratified block randomization method using ‘Frequency Scale for the Symptoms of Gastroesophageal Reflux Disease’ (FSSG), ‘Gastrointestinal Symptoms Rating Scale’ (GSRS), age, and sex as allocation adjustment factors. The controller assigned the two groups (placebo and LJ88 groups). The allocation table had been sealed by the controller and kept sealed until the allocation table was to be opened after the termination of all data collection. A flow diagram of the study is shown in Figure 2.

### 2.2. Participants

We used the FSSG [19,20] and the Rome IV classification for functional dyspepsia [21] to investigate the effects of heat-killed LJ88 in healthy adults with mild and temporal gastroesophageal reflux-related symptoms. Participants were selected if they had a FSSG total score ≥ 8 (indicating the presence of gastroesophageal reflux-related symptoms) and if they displayed none of the diagnostic criteria for functional dyspepsia B1a (bothersome postprandial fullness; bothersome early satiation; bothersome epigastric pain; bothersome epigastric burning) within the last 3 months, nor had they been onset within the last 6 months (indicating that they did not have functional dyspepsia, and that their gastroesophageal reflux-related symptoms were temporal and not bothersome). To omit participants with gastric symptoms related to *Helicobacter pylori* infection, candidates were screened with the *Helicobacter pylori* antibody test, and participants were selected only if the anti-*Helicobacter pylori* antibody titer was lower than 10 units/mL.

Participants were included if they: (1) were aged between 20 and 64 years on the date of obtaining consent, (2) had a FSSG total score ≥ 8, and (3) had received sufficient explanation of the purpose and contents of the research, had the ability to consent, fully understood and voluntarily participated in the research, and consented to participate in this study in writing. Participants were excluded if they: (1) had a history of *H. pylori* infection or tested positivity for *Helicobacter pylori* antibodies (blood anti-*Helicobacter pylori* antibodies), (2) regularly used drugs that affect stomach symptoms, (3) had been diagnosed with functional dyspepsia according to the ROME IV classification (specifically, those who had upper abdominal symptoms that felt painful for more than 6 months and had symptoms for the past 3 months), (4) were unable to restrict the intake of probiotic foods, prebiotic foods, foods containing lactic acid bacteria and bifidobacteria, or other health foods that were effective in improving gastric symptoms during the study period, (5) had existing medical conditions, including organic diseases of the stomach (gastric ulcer, gastric cancer, gastritis, and gastroesophageal reflux disease), (6) had food allergies, (7) had frequently become aware of not feeling well because of dairy product intake, (8) had diseases requiring urgent treatment or with serious complications, (9) had gastrointestinal diseases that affect digestion and absorption or defecation or those with a history of gastrointestinal surgery, (10) were judged unsuitable as research subjects based on the blood tests performed during the screening test, (11) were pregnant, intending to become pregnant during the research period, or were breastfeeding, (12) had a history of drug dependence or alcohol dependence or current illness, (13) were participating or intended to participate in research that used other foods or drugs or applied cosmetics or drugs, (14) were judged by the principal investigator as inappropriate research participants. Participants were advised to maintain a daily diary of their health conditions, whether or not the test food was consumed and the time of its intake, use of medicines, other changes in physical condition mainly gastrointestinal symptoms, changes in living conditions, the contents of all foods consumed, etc., throughout the study period. Based on the diary, the compliance of the participants was assessed.

An outline of the participant selection process is shown in Figure 1. First, 302 candidates were selected for screening from over 1000 registered volunteers, based on the results of questionnaires regarding the use of drugs and supplements, dietary habits, alcohol consumption, food allergies, and the FSSG. The screening tests consisted of doctor interviews involving assessment of the ROME IV diagnostic criteria for functional dyspepsia B1a, somatometry, blood pressure, pulse rate, *Helicobacter pylori* antibody test, blood cellular test, blood biochemical tests, urinalysis, and the GSRS. We then enrolled 179 participants who had been selected from the 302 candidates for the first round ‘ingestion of placebo period’. Finally, based on the extent of placebo effects, degree of compliance to instructions, and results of other test items, 120 participants, aged 21–63 years, were selected for the second round ’ingestion of test foods period’. As the results, all 120 participants were without any underlying diseases and medication. The final participants were considered healthy because their gastroesophageal reflex-related symptoms were mild and temporal, and they had no underlying diseases or medications.

The sample size was based on the results of the previous pilot clinical study [18], where the change in FSSG total score after 6-weeks of ingestion of 1 billion cells of heat-killed LJ88 ranged from 6.79 ± 5.35 to 3.00 ± 2.80 (55.8% decrease). Assuming the placebo effect to be 20%, the FSSG total scores after 6-weeks ingestion of the test food in the placebo and LJ88 groups were calculated to be 5.43 ± 5.35 and 3.00 ± 2.80, respectively. Based on the Cohen’s method [22], the effect size (d) was calculated to be d = 0.5695808, and by assuming α-error and detection power to be 0.05 and 0.8, respectively, the sample size for each group was calculated to be 50 (total = 100). The final number of participants in each group was set to be 60 (total = 120), to account for a potentially higher placebo effect, intermediate dropout, post-experiment exclusion.

### 2.3. Test Foods

A daily sachet containing 1 billion cells of LJ88 was used as the test food for the LJ88 group. LJ88 cells were cultured in a stirred-tank fermenter, washed with water, mixed with dextrin, heat-inactivated, and spray dried. Each sachet contained 1.2 g of powder ingredients was composed of 0.598 g granulated isomalt, 0.35 g β-cyclodextrin, 0.2 g D-sorbitol, 0.012 g sucrose stearate, 0.02 g dextrin, and the LJ88 raw material containing heat-killed LJ88 and dextrin (0.02 g). Granulated isomalt was used for the placebo food instead of 0.02 g of LJ88 raw material containing heat-killed LJ88, whereas other ingredients were the same as the test food for LJ88 group, so that both test foods could not be discriminated by their tastes and appearances. Participants were instructed, as a general rule, to take one sachet once a day before going to bed with water or lukewarm water.

### 2.4. Measurements

#### 2.4.1. Primary Endpoint

The FSSG scoring system proposed by Kusano et al. was used as the primary endpoint [19,20]. This system consists of 12 questions rated on a 5-point scale (never, occasionally, sometimes, often, and always), and scores of 0 to 4 are assigned to each question. The 12 questions are as follows: 1. Do you get heartburn? 2. Does your stomach get bloated? 3. Does your stomach ever feel heavy after meals? 4. Do you sometimes subconsciously rub your chest with your hand? 5. Do you ever feel sick after meals? 6. Do you get heartburn after meals? 7. Do you have an unusual (e.g., burning) sensation in your throat? 8. Do you feel full while eating meals? 9. Do some things get stuck when you swallow? 10. Do you get bitter liquid (acid) coming up into your throat? 11. Do you burp a lot? 12. Do you get heartburn if you bend over? The effects of heat-killed LJ88 on gastroesophageal reflux-related symptoms were evaluated using the score for each question, two sub-scores for acid reflux (sum of 1, 4, 6, 7, 9, 10, and 12) and acid-related dyspeptic (sum of 2, 3, 5, 8, and 11) symptoms, and the total score (sum of all scores).

#### 2.4.2. Secondary Endpoints

The GSRS [23] was used to evaluate the presence of gastrointestinal symptoms. The GSRS scale consists of 15 items answered on a scale of 4 (0, 1, 2, and 3, indicating lighter to heavier symptoms). The 15 items are: 1. Abdominal pain; 2. Heartburn; 3. Acid regurgitation; 4. Sucking sensations in the epigastrium; 5. Nausea and vomiting; 6. Borborygmus; 7. Abdominal distension; 8. Eructation; 9. Increased flatus; 10. Decreased passage of stools; 11. Increased passage of stools; 12. Loose stools; 13. Hard stools; 14. Urgent need for defecation; 15. Feeling of incomplete evacuation. Individual item scores and the total sum for all items were used to evaluate the effects of the test foods.

Subjective feelings of improvement in the stomach state were measured at 3- and 6-weeks using a single question, “Was your stomach state improved by ingestion of the test food?” using a 5-point response scale: 1. Very improved; 2. Slightly improved; 3. No change; 4. Slightly worsened; 5. Very worsened.

To examine the potential role of gastrin as an underlying mechanism, serum gastrin concentration was measured using a radioimmunoassay method (Gastrin RIA kit DP, DENIS Pharma K.K., Tokyo, Japan) at 0 and 6 weeks after ingestion of the test foods. As the lower quantification limit was 15 pmol/mL, concentrations below 15 pmol/mL were assumed to be 14 pmol/mL for statistical analyses.

As the state of the stomach has been shown to be related to psychological state and overall quality of life, psychological symptoms and subjective quality of life were evaluated using the Japanese edition of ‘The Profile of Mood States, second edition’ (POMS2) [24,25] and the ‘36-item Short-Form Health Survey (SF-36) v2’ [26,27], respectively.

#### 2.4.3. Blood Tests and Urinalysis

Blood biochemical tests, blood cellular tests, and urinalysis were performed at visit 1 and visit 4. The blood biochemical tests assessed aspartate aminotransferase (AST), alanine aminotransferase (ALT), lactate dehydrogenase (LD: IFCC), total bilirubin, alanine aminotransferase (ALT), γ-glutamyltransferase (γ-GT), glucose, HbA1c (NGSP), total cholesterol, low-density lipoprotein (LDL) cholesterol, high-density lipoprotein (HDL) cholesterol, triglyceride (TG), total protein, albumin, urea nitrogen, creatinine, uric acid, sodium (Na), chloride (Cl), potassium (K), and calcium (Ca) levels. The blood cellular tests assessed blood cell count, red blood cell count, hemoglobin content, hematocrit, platelet count, mean corpuscular volume (MCV), mean corpuscular hemoglobin (MCH), mean corpuscular hemoglobin concentration (MCHC), and leukocyte counts (neutrophils, lymphocytes, monocytes, eosinophils, and basophils). Urinalysis involved the measurement of pH, specific gravity, urine protein, glucose, urobilinogen, bilirubin, ketone bodies, and urinary occult blood reactions. Bood was withdrawn and urine was taken under the following condition: (1) no alcohol from the day before the test, (2) fasting from 21:00 on the day before the test until the end of the test (but water may be consumed), and (3) no smoking from the time of waking on the day of the test.

### 2.5. Statistical Analysis

Statistical comparisons between groups at 0, 3 and 6 weeks were performed using an unpaired Student’s *t*-test or Wilcoxon rank sum test. Statistical comparisons between 2 time points (0 and 6 weeks) were performed using Student’s *t*-test (paired) or Wilcoxon signed rank test. Statistical comparison between 3 time points (0, 3, and 6 weeks) were performed using Dunnett’s test (paired) or Wilcoxon signed rank test with Bonferroni correction of multiplicity. Statistical analysis of the rate of occurrence data, including age and relief rate, was performed using Fisher’s exact test. The statistical significance level was set at 0.05. Data were analyzed using IBM SPSS Statistics ver.24.

## 3. Results

### 3.1. Baseline Characteristics of Participants

Table 1 summarizes the baseline characteristics of the participants assigned to each group (n = 60 in each group; full analysis set, FAS). There were no significant differences in sex ratio, age, height, body weight, body mass index (BMI), systolic and diastolic blood pressures, pulse rate, and total FSSG score between the groups. Similarly, there were no significant differences between groups in the results of blood cellular analysis, blood biochemical analysis, and urinalysis (Table S1). These results show that randomization of participants across the two groups was well balanced.

### 3.2. The Effect of Heat-Killed LJ88 on Gastroesophageal Reflux-Related Symptoms

#### 3.2.1. FSSG

**Figure 3:**
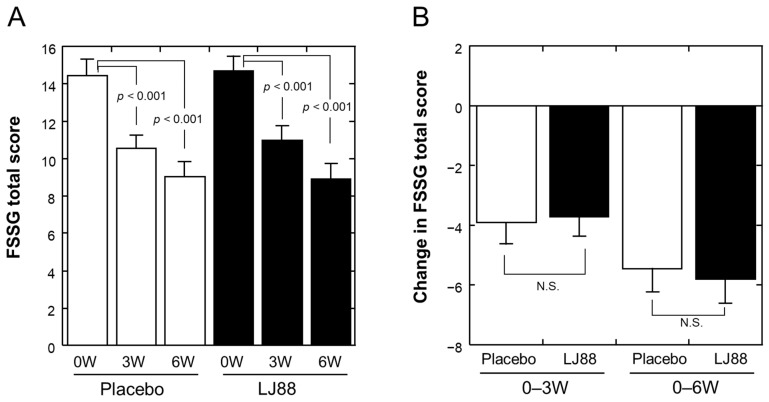
(**A**) FSSG total score at 0 (baseline), 3, and 6 weeks of ingestion of test foods. (**B**) Change in FSSG total score from 0 to 3 weeks (0–3 W) and 0 to 6 weeks (0–6 W). Open and closed bars represent placebo and LJ88 groups, respectively. Means with standard errors are plotted. The significant probabilities are shown in the Figures, and N.S. denotes “not significant”.

**Figure 4:**
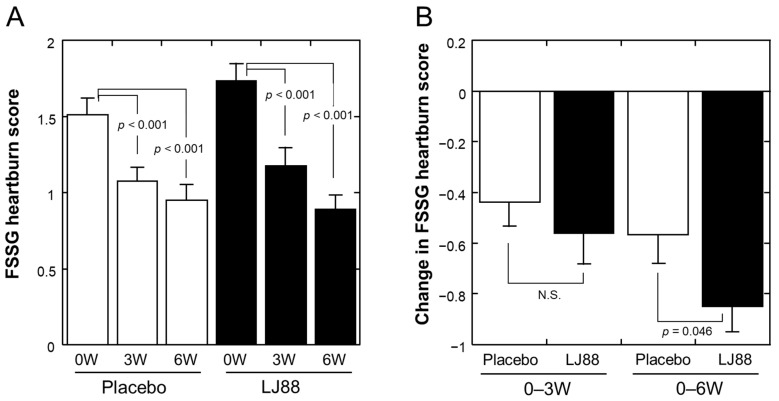
(**A**) FSSG heartburn score at 0 (baseline), 3, and 6 weeks of ingestion of test foods. (**B**) Change in FSSG heartburn score from 0 to 3 weeks (0–3 W) and 0 to 6 weeks (0–6 W). Open and closed bars represent placebo and LJ88 groups, respectively. Means with standard errors are plotted. The significant probabilities are shown in the Figures, and N.S. denotes “not significant”.

Table 2 summarizes the effects of test foods on FSSG scores (n = 55 and 52 for the placebo and LJ88 groups, respectively; per protocol set, PPS). The total score, sub-scores for acid reflux-related and dyspeptic symptoms, and almost all specific scores of the FSSG questionnaire were significantly improved at 3 and 6 W compared to baseline (0 W) in both groups. The changes in total and heartburn scores are depicted in Figure 3A and Figure 4A, respectively, as well. The FSSG score for the item “1. Do you get heartburn?” was significantly improved at 6 W in the LJ88 group compared to the placebo group (*p* = 0.046; Figure 4B), whereas total score (Figure 3B) and other specific and sub-scores did not (Table 2). Conversion of the FSSG data to relief rates between 0 and 6 W (Table 3) indicated a significantly higher relief rate in the LJ88 group than in the placebo group (*p* = 0.049).

#### 3.2.2. GSRS

The score for two GSRS items related to acid reflux (2. Heartburn; 3. Acid regurgitation) was improved at 3 and 6 W compared to baseline in both groups (Table 4). In addition, the score for two other items (4. Sucking sensation in the epigastrium; 7. Abdominal distension) and the total score were improved at 6 W compared to baseline in both groups. The change in GSRS score between baseline and 3 W for one item (5. Nausea and vomiting) was significantly different between groups (*p* = 0.029), with slight improvement and worsening of this symptom in the placebo and LJ88 groups, respectively. However, this difference was not observed at 6 W. Conversion of the GSRS data to relief rates (Table 5) showed no significant differences between the groups for all items and at all time points.

#### 3.2.3. Stomach State Questionnaire

No statistical differences were observed in the subjective improvement of the stomach between the groups, as indicated by the score and relief rates (Table 6).

#### 3.2.4. Serum Gastrin Concentration

Serum gastrin concentrations at 6 W were not significantly different from those at baseline in either group, and no significant differences in concentration were observed between groups (Table 7).

### 3.3. Psychological Symptoms and Quality of Life

#### 3.3.1. POMS2

The psychological symptom ‘Friendliness’ measured using the POMS2 was significantly worse at 6 W compared to baseline in the placebo group (Table 8). However, no significant differences were observed between the groups in the change in scores from baseline to 6 W for all items.

#### 3.3.2. SF36v2

**Figure 5:**
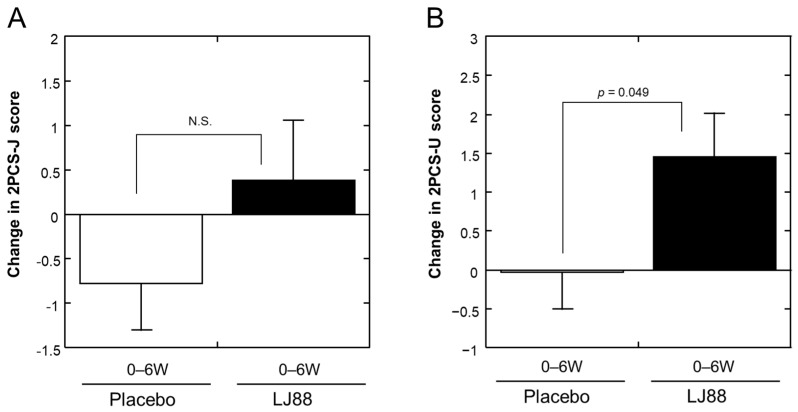
Changes from 0 to 6 weeks (0–6 W) in (**A**) physical component summary Japanese version (two components; 2PCS-J) and (**B**) physical component summary universal version (two components; 2PCS-U) of SF-36v2 scores. Open and closed bars represent placebo and LJ88 groups, respectively. Means with standard errors are plotted. The significant probabilities are shown in the Figures, and N.S. denotes “not significant”.

As shown in Table 9, two quality of life items as measured using the SF36v2 (Physical Component Summary (three components); Physical Component Summary Universal (2 components)) increased significantly in the LJ88 group, but not in the placebo group, between 0 W and 6 W (*p* = 0.029 and 0.014, respectively). Similarly, a significant difference between the groups was observed in the change (from 0 to 6 W) in the Physical Component Summary Universal (two components) (*p* = 0.049; Figure 5B), although the change in the Physical Component Summary (three components) was only marginally significant (*p* = 0.050). Although this study was conducted with Japanese participants, no statistical significance was observed in the score of the physical component using the Japanese version of the instrument (Physical Component Summary Japanese (two components)) (Figure 5A).

### 3.4. Safety Aspects

During the study, adverse events were observed in 24 of the 120 participants (13 and 11 in the placebo and LJ88 groups, respectively), and the total number of events was 38 (19 and 19 in the placebo and LJ88 groups, respectively). In the placebo group, the 19 adverse events were sore throat (one case), cold (one case), high glucose (one case), swelling and pain in both eyelids (one case), pollen allergy (three cases), stomach ache (two cases), periodontal disease (one case), postmenopausal symptoms (two cases), thumb cut (one case), tired eyes (four cases), fatigue (one case), and nasal mucus (one case). In the LJ88 group, the 19 adverse events were diarrhea (one case), heavy stomach (one case), nausea (one case), malaise (one case), unwellness via stress (one case), fatigue (five cases), abdominal bloating and too much gas (one case), high triglyceride (one case), dry eye (one case), cold (one case), pollen allergy (one case), tired eye (two cases), headache (one case), and COVID-19 (one case). None of these factors, however, was considered serious or related to the ingestion of either test food. No abnormal changes were detected in body weight, BMI, blood pressure, pulse rate (Table S2), blood biochemical test values (Table S3), blood cellular test results (Table S4), or urinalysis results (Table S5), although some statistically significant changes within normal ranges were observed for some items in both groups. In summary, these results suggest that both test foods (placebo and LJ88) were safe.

## 4. Discussion

This randomized, placebo-controlled, double-blind, parallel-group clinical study investigated whether the daily ingestion of heat-killed LJ88 had beneficial effects on temporal gastroesophageal reflux-related symptoms in healthy Japanese volunteers. Although no statistically significant differences were observed between the groups in the total FSSG score and almost all of the secondary endpoint scores, the heartburn score of the FSSG was significantly improved at 6 W in the LJ88 group compared to that in the placebo group (Table 2 and Table 3; Figure 4B), indicating that the daily ingestion of heat-killed LJ88 at a dose of 1 billion cells/day for 6 weeks can improve temporal heartburn symptoms related to gastroesophageal reflux. To the best of our knowledge, this is the first report demonstrating beneficial effects of non-living lactic acid bacteria on heartburn symptoms. In addition, significant improvement in the score for the Physical Component Summary_Universal (two components) of the SF-36v2 after 6-weeks of ingestion of heat-killed LJ88-containing test food compared to the placebo (Table 9 and Figure 5B) indicates a beneficial effect of heat-killed LJ88 on the physical aspects of quality of life. This latter finding would need to be confirmed by further study, because the Japanese version of the physical component summary did not significantly improve in the LJ88 group compared to the placebo group (Figure 5A), despite the participants in this study all being Japanese.

Some specific scores in the FSSG (Table 2) and some gastroesophageal reflux-related scores in the GSRS (Table 4) were significantly improved at 3 and/or 6 W compared to baseline in both groups. These changes may reflect the placebo effect. Similar changes in FSSG and GSRS scores were also observed in a clinical study on the effects on gastrointestinal discomfort and symptoms of *Bifidobacterium bifidum* YIT20347, although statistical analyses of the time-dependent changes were not performed [7]. A relatively strong placebo effect on heartburn frequency was also reported in a clinical study with soy fermentation [28]. These changes suggest a strong relationship between the stomach and brain, such that mental preparation for participation in the clinical study to examine the effects on the stomach can cause anticipated positive changes in the stomach. Another possibility is that individuals with only mild and temporal discomfort in the stomach were selected for inclusion in the study, and therefore it is reasonable to expect that the symptoms would naturally heal in a relatively short period. However, even under such probably high placebo effects and/or natural healing biases, heartburn symptoms were significantly improved at 6 W in the LJ88 group compared to the placebo group in our study.

As mentioned above, no statistically significant differences were observed between the groups in the total FSSG score. However, the total scores of the placebo group at 0 and 6 W were 14.42 and 8.98, respectively, indicating that the placebo effect and/or natural healing trend was 37.7%, which was nearly double the assumed placebo effect of 20%, based on the pilot clinical study [18]. Furthermore, the improvement in the LJ88 group was 39.5% (from 14.65 to 8.87), which was smaller than that reported (55.8%) in the pilot clinical study. This may be the reason for the lack of a significant difference between the groups in the FSSG total score.

A decrease in serum gastrin concentration has been proposed as part of the mechanism underlying the effect of LJ88 in improving the hyper acidic condition of the stomach, based on in vivo animal studies [14] and the pilot clinical study [18]. In the current study, however, serum gastrin concentration was not significantly decreased by ingestion of the LJ88-containing test food, and there was no significant difference between groups in the change in serum gastrin concentration from 0 W to 6 W. Baseline serum gastrin concentrations before ingestion of test foods in 105 out of 107 PPS participants were below 46.9 pmoles/L, which is considered to be within the normal range [29]. However, in two participants who were both in the LJ88 group, baseline serum gastrin concentrations were 264 and 53 pmoles/L, which is higher than the upper limit of the normal range. Corresponding serum gastrin concentrations at 6 W were decreased to 212 and 24 pmol/L, respectively. Therefore, although most of the temporal gastroesophageal reflux-related symptoms in the participants in this study were seemingly not related to high serum gastrin levels, in some participants, a decrease in serum gastrin concentration possibly reduced their stomach complications. The underlying mechanisms, other than gastrin regulation, for improving temporal gastroesophageal reflux-related symptoms such as heartburn, remain unclear. Heat-killed LJ88 has been shown to have anti-*H. pylori* activity in vitro and in vivo. However, since all participants in this study were *H. pylori* antibody-negative and had no history of *H. pylori* infection, the effect of heat-killed LJ88 on temporal heartburn could not be explained by anti-*H. pylori* effects. 

The limitations to this clinical study include: (1) a relatively high placebo effect and/or a natural healing trend; (2) a decrease in serum gastrin concentration could not be confirmed as a possible underlying mechanism; and (3) although significant improvement in the FSSG heartburn score in the LJ88 group compared with the placebo group was confirmed, the total FSSG score was not. These limitations may be related to the close relationship between the stomach and the brain [30]. The subjective condition of the stomach is possibly highly related to the mental states of the subjects; conversely, the mental states of the subjects might affect their stomach conditions. In fact, psychological approaches have been proposed in addition to pharmaceutical ones to treat gastroesophageal reflux diseases and functional esophageal disorders [4,31,32]. Future studies should aim to minimize the effects of such psychological factors. This may be achieved by the recruitment of participants with more severe stomach symptoms than in this study; for example, participants with higher FSSG scores and/or higher serum gastrin concentrations. Such a study may more clearly discriminate the effect of the test food from the placebo and to bring about a larger decrease in serum gastrin concentrations. Another method may be to examine the effects of LJ88 under controlled stress conditions. Stress situations may induce more stomach discomfort symptoms in subjects, resulting in a clearer effect of the test foods. One study showed that the ingestion of fermented milk containing the probiotic *Lactobacillus casei* strain Shirota relieved stress-associated symptoms, including abdominal dysfunction, in healthy medical students under academic examination stress [33]. Vagal afferent signaling to the brain is suggested to contribute to the anti-stress effect [34]. Since, in animal studies, heat-killed LJ88 was reported to increase the number of bifidobacteria in the feces, suggesting the improvement of gut microbiota [17], the signaling from the gut to the brain might possibly play a role as an underlying mechanism of heat-killed LJ88 to improve FSSG heartburn score, as in the *Lactobacillus casei* strain Shirota mentioned above. Therefore, in the future study, the analysis of microbiota in the feces of participants as the objective parameter, and questionnaires targeting more to the state of the gut as the subjective parameter, would be more appropriate. Although other underlying mechanisms of heat-killed LJ88 to improve FSSG heartburn score might possibly exist, e.g., the direct effect on the stomach not related to gastrin and via improvement of physical component of quality of life, further studies will be necessary to elucidate them. Moreover, in this study, we employed a large number of different measurements, which possibly raises the problem of multiplicity in statistics. However, in this paper, we did not explicitly take this problem into consideration. This is another limitation of this study.

## 5. Conclusions

This randomized, placebo-controlled, double-blind, parallel-group clinical study indicated that the daily ingestion of heat-killed LJ88 (1 billion cells/day) for 6 weeks has beneficial effects on the temporal heartburn symptoms related to gastroesophageal reflux, and has no safety concerns. This effect was accompanied by an improvement in the physical aspects of quality of life.

## Figures and Tables

**Table 1:** Baseline characteristics of the participants (full analysis set).

Items		All Participants (n = 120)			Placebo Group (n = 60)			LJ88 Group (n = 60)			*p*-Value	(Method) ^(1)^
Sex	female	67			33			34			1.000	(Fisher’s exact test)
	male	53			27			26				
Age (Years)		46.7	±	10.2	46.9	±	9.7	46.5	±	10.7	0.817	(Student’s *t*-test)
		(21–63) ^(2)^			(23–63) ^(2)^			(21–63) ^(2)^				
Height (cm)		164.6	±	8.4	164.7	±	8.7	164.6	±	8.1	0.922	
Body weight (kg)		60.4	±	11.5	60.6	±	11.5	60.3	±	11.5	0.889	
BMI		22.2	±	3.2	22.2	±	3.1	22.1	±	3.3	0.887	
Systolic blood pressure (mmHg)		116.9	±	14.1	119.2	±	15.2	114.7	±	12.7	0.086	
Diastolic blood pressure (mmHg)		72.5	±	10.1	73.0	±	10.9	72.1	±	9.4	0.629	
Pulse rate (bpm)		69.0	±	8.8	68.4	±	8.5	69.7	±	9.2	0.434	
FSSG total score		22.2	±	5.8	22.0	±	5.4	22.4	±	6.2	0.805	(Wilcoxon rank sum test)
		(14–47) ^(2)^			(15–35) ^(2)^			(14–47) ^(2)^				

Mean ± SD or number of participants are depicted. ^(1)^ Methods for statistical analyses. ^(2)^ Range of the values (lowest–highest).

**Table 2:** Change in FSSG scores (per-protocol set).

Items	Group	n	FSSG Scores											Change in FSSG Scores							
			0 W			3 W				6 W				0–3 W				0–6 W			
			Mean	±	SD	Mean	±	SD	*p* Value ^(1)^	Mean	±	SD	*p* Value ^(1)^	Mean	±	SD	*p* Value ^(2)^	Mean	±	SD	*p* Value ^(2)^
1. Do you get heartburn?	>Placebo	>55	1.51	±	0.79	1.07	±	0.63	0.000	0.95	±	0.73	0.000	−0.44	±	0.71	0.334	−0.56	±	0.86	0.046
	LJ88	52	1.73	±	0.82	1.17	±	0.86	0.000	0.88	±	0.65	0.000	−0.56	±	0.89		−0.85	±	0.72	
2. Does your stomach get bloated?	Placebo	55	1.62	±	0.89	1.31	±	0.90	0.002	1.20	±	0.97	0.003	−0.31	±	0.66	0.305	−0.42	±	0.94	0.209
	LJ88	52	1.92	±	0.99	1.48	±	0.96	0.002	1.23	±	0.90	0.000	−0.44	±	0.92		−0.69	±	0.96	
3. Does your stomach ever feel heavy after meals?	Placebo	55	1.78	±	0.74	1.27	±	0.73	0.000	1.07	±	0.81	0.000	−0.51	±	0.84	0.075	−0.71	±	0.63	0.199
	LJ88	52	1.69	±	0.81	1.40	±	0.85	0.016	1.23	±	0.67	0.002	−0.29	±	0.82		−0.46	±	0.94	
4. Do you sometimes subconsciously rub your chest with your hand?	Placebo	55	0.82	±	0.84	0.53	±	0.69	0.001	0.40	±	0.60	0.000	−0.29	±	0.60	0.896	−0.42	±	0.79	0.209
	LJ88	52	0.94	±	0.87	0.69	±	0.85	0.029	0.37	±	0.63	0.000	−0.25	±	0.79		−0.58	±	0.78	
5. Do you ever feel sick after meals?	Placebo	55	1.15	±	0.99	0.73	±	0.62	0.002	0.64	±	0.75	0.001	−0.42	±	0.92	0.107	−0.51	±	1.00	0.614
	LJ88	52	1.12	±	0.92	1.00	±	0.74	0.419	0.71	±	0.72	0.003	−0.12	±	0.92		−0.40	±	0.89	
6. Do you get heartburn after meals?	Placebo	55	1.49	±	0.90	1.02	±	0.73	0.001	0.82	±	0.72	0.000	−0.47	±	0.92	0.509	−0.67	±	0.86	0.812
	LJ88	52	1.50	±	0.87	1.13	±	0.74	0.001	0.83	±	0.76	0.000	−0.37	±	0.69		−0.67	±	0.76	
7. Do you have an unusual (e.g., burning) sensation in your throat?	Placebo	55	0.65	±	0.87	0.47	±	0.77	0.116	0.25	±	0.62	0.001	−0.18	±	0.82	0.335	−0.40	±	0.78	0.051
	LJ88	52	0.42	±	0.85	0.33	±	0.71	0.519	0.25	±	0.71	0.132	−0.10	±	0.87		−0.17	±	0.90	
8. Do you feel full while eating meals?	Placebo	55	1.20	±	1.03	0.96	±	0.96	0.051	0.96	±	1.04	0.104	−0.24	±	0.86	0.760	−0.24	±	1.00	0.482
	LJ88	52	1.23	±	1.02	0.94	±	0.92	0.024	0.90	±	1.01	0.018	−0.29	±	0.87		−0.33	±	0.98	
9. Do some things get stuck when you swallow?	Placebo	55	0.80	±	0.93	0.55	±	0.72	0.037	0.44	±	0.66	0.008	−0.25	±	0.87	0.783	−0.36	±	0.97	0.590
	LJ88	52	0.69	±	0.81	0.40	±	0.66	0.008	0.33	±	0.73	0.003	−0.29	±	0.75		−0.37	±	0.91	
10. Do you get bitter liquid (acid) coming up into your throat?	Placebo	55	1.09	±	0.91	0.78	±	0.71	0.006	0.60	±	0.71	0.000	−0.31	±	0.79	0.891	−0.49	±	0.81	0.774
	LJ88	52	1.00	±	0.79	0.73	±	0.72	0.008	0.60	±	0.77	0.000	−0.27	±	0.69		−0.40	±	0.66	
11. Do you burp a lot?	Placebo	55	1.45	±	1.07	1.11	±	0.96	0.002	1.11	±	0.98	0.003	−0.35	±	0.78	0.735	−0.35	±	0.80	0.265
	LJ88	52	1.60	±	0.93	1.17	±	0.86	0.001	1.10	±	0.91	0.000	−0.42	±	0.78		−0.50	±	0.75	
12. Do you get heartburn if you bend over?	Placebo	55	0.85	±	1.01	0.73	±	0.91	0.231	0.55	±	0.86	0.009	−0.13	±	0.86	0.124	−0.31	±	0.81	0.377
	LJ88	52	0.81	±	0.74	0.50	±	0.70	0.005	0.44	±	0.73	0.002	−0.31	±	0.73		−0.37	±	0.84	
Total score	Placebo	55	14.42	±	6.24	10.53	±	4.89	0.000	8.98	±	5.77	0.000	−3.89	±	5.32	0.710	−5.44	±	5.71	0.376
	LJ88	52	14.65	±	5.75	10.96	±	5.63	0.000	8.87	±	6.10	0.000	−3.69	±	4.74		−5.79	±	5.78	
Sub-score: Acid reflux related symptom (1, 4, 6, 7, 9, 10, 12)	Placebo	55	7.22	±	4.22	5.15	±	2.85	0.000	4.00	±	3.18	0.000	−2.07	±	3.34	0.466	−3.22	±	3.73	0.248
	LJ88	52	7.10	±	3.60	4.96	±	3.27	0.000	3.69	±	3.67	0.000	−2.13	±	2.70		−3.40	±	3.34	
Sub-score: Dyspeptic (Dysmotility) symptom (2, 3, 5, 8, 11)	Placebo	55	7.20	±	2.83	5.38	±	2.74	0.000	4.98	±	3.27	0.000	−1.82	±	2.53	0.639	−2.22	±	2.61	0.706
	LJ88	52	7.56	±	2.75	6.00	±	2.89	0.000	5.17	±	2.98	0.000	−1.56	±	2.55		−2.38	±	2.96	

^(1)^ Wilcoxon signed-rank test (vs. 0 W) with Bonferroni correction. ^(2)^ Wilcoxon rank sum test (between groups).

**Table 3:** FSSG relief rate (per-protocol set).

Items	Group	n	Relief Rate ^(1)^					
			0 W to 3 W			0 W to 6 W		
			Not Alleviated	Alleviated	*p* Value ^(2)^	Not Alleviated	Alleviated	*p* Value ^(2)^
1. Do you get heartburn?	Placebo	55	32	23	0.178	28	27	0.049
	LJ88	52	23	29		16	36	
2. Does your stomach get bloated?	Placebo	55	36	19	0.173	31	24	0.248
	LJ88	52	27	25		23	29	
3. Does your stomach ever feel heavy after meals?	Placebo	55	28	27	0.076	19	36	0.242
	LJ88	52	36	16		24	28	
4. Do you sometimes subconsciously rub your chest with your hand?	Placebo	55	41	14	0.667	33	22	0.177
	LJ88	52	36	16		24	28	
5. Do you ever feel sick after meals?	Placebo	55	33	22	0.690	29	26	0.698
	LJ88	52	34	18		30	22	
6. Do you get heartburn after meals?	Placebo	55	27	28	0.563	25	30	0.557
	LJ88	52	29	23		20	32	
7. Do you have an unusual (e.g., burning) sensation in your throat?	Placebo	55	39	16	0.061	35	20	0.056
	LJ88	52	45	7		42	10	
8. Do you feel full while eating meals?	Placebo	55	34	21	1.000	36	19	0.843
	LJ88	52	33	19		33	19	
9. Do some things get stuck when you swallow?	Placebo	55	38	17	1.000	36	19	0.693
	LJ88	52	35	17		32	20	
10. Do you get bitter liquid (acid) coming up into your throat?	Placebo	55	40	15	0.673	30	25	1.000
	LJ88	52	35	17		28	24	
11. Do you burp a lot?	Placebo	55	32	23	1.000	32	23	0.336
	LJ88	52	31	21		25	27	
12. Do you get heartburn if you bend over?	Placebo	55	43	12	0.059	38	17	0.235
	LJ88	52	31	21		30	22	
Total score	Placebo	55	14	41	1.000	7	48	1.000
	LJ88	52	13	39		7	45	
Sub-score: Acid reflux related symptom (1, 4, 6, 7, 9, 10, 12)	Placebo	55	20	35	0.217	11	44	0.617
	LJ88	52	13	39		8	43	
Sub-score: Dyspeptic (Dysmotility) symptom (2, 3, 5, 8, 11)	Placebo	55	20	35	0.844	14	41	0.824
	LJ88	52	20	32		12	40	

^(1)^ Relief rate is the number of participants whose symptoms were alleviated or not at 3 W or 6 W compared to 0 W based on the changes in FSSG scores. ^(2)^ Fisher’s exact test.

**Table 4:** Change in GSRS scores (per-protocol set).

Items	Group	n	GSRS Scores											Change in GSRS Scores							
			0 W			3 W				6 W				0–3 W				0–6 W			
			Mean	±	SD	Mean	±	SD	*p* Value ^(1)^	Mean	±	SD	*p* Value ^(1)^	Mean	±	SD	*p* Value ^(2)^	Mean	±	SD	*p* Value ^(2)^
1. Abdominal pain	Placebo	55	1.98	±	0.93	1.89	±	0.79	0.382	1.84	±	0.92	0.254	−0.09	±	0.80	0.728	−0.15	±	0.91	0.260
	LJ88	52	2.12	±	0.92	1.96	±	0.86	0.166	1.81	±	0.77	0.029	−0.15	±	0.83		−0.31	±	0.94	
2. Heartburn	Placebo	55	2.24	±	1.12	1.96	±	0.82	0.024	1.58	±	0.83	0.000	−0.27	±	0.87	0.777	−0.65	±	1.06	0.778
	LJ88	52	2.37	±	0.89	2.06	±	0.96	0.010	1.73	±	0.74	0.000	−0.31	±	0.81		−0.63	±	0.82	
3. Acid regurgitation	Placebo	55	2.11	±	1.05	1.87	±	0.86	0.045	1.67	±	0.86	0.003	−0.24	±	0.86	0.723	−0.44	±	1.03	0.857
	LJ88	52	2.00	±	0.84	1.77	±	0.81	0.037	1.63	±	0.71	0.001	−0.23	±	0.76		−0.37	±	0.71	
4. Sucking sensations in the epigastrium	Placebo	55	2.13	±	1.06	1.96	±	1.00	0.112	1.82	±	1.00	0.012	−0.16	±	0.76	0.784	−0.31	±	0.86	0.554
	LJ88	52	2.15	±	1.06	1.98	±	0.98	0.130	1.71	±	0.78	0.002	−0.17	±	0.81		−0.44	±	0.94	
5. Nausea and vomiting	Placebo	55	1.58	±	0.69	1.42	±	0.79	0.044	1.31	±	0.50	0.005	−0.16	±	0.83	0.029	−0.27	±	0.68	0.081
	LJ88	52	1.42	±	0.80	1.50	±	0.75	0.475	1.37	±	0.60	0.826	0.08	±	0.88		−0.06	±	0.85	
6. Borborygmus	Placebo	55	2.40	±	1.18	2.40	±	1.23	0.973	2.33	±	1.28	0.442	0.00	±	1.11	0.801	−0.07	±	0.90	0.493
	LJ88	52	2.42	±	1.07	2.44	±	1.23	0.769	2.15	±	1.18	0.059	0.02	±	0.90		−0.27	±	0.95	
7. Abdominal distension	Placebo	55	2.42	±	1.08	2.16	±	1.12	0.105	2.07	±	1.02	0.021	−0.25	±	1.13	0.896	−0.35	±	1.04	0.256
	LJ88	52	2.65	±	1.08	2.42	±	1.11	0.128	2.10	±	1.01	0.000	−0.23	±	1.06		−0.56	±	0.98	
8. Eructation	Placebo	55	2.36	±	1.25	2.13	±	1.06	0.057	2.00	±	1.12	0.009	−0.24	±	0.90	0.891	−0.36	±	0.97	0.736
	LJ88	52	2.33	±	1.06	2.17	±	0.98	0.208	1.98	±	0.92	0.005	−0.15	±	0.85		−0.35	±	0.84	
9. Increased flatus	Placebo	55	2.76	±	1.14	2.75	±	1.28	0.990	2.53	±	1.23	0.082	−0.02	±	0.97	0.957	−0.24	±	1.05	0.745
	LJ88	52	3.02	±	1.09	2.94	±	1.21	0.720	2.69	±	1.29	0.064	−0.08	±	1.01		−0.33	±	1.15	
10 Decreased passage of stools	Placebo	55	2.00	±	1.07	2.04	±	1.14	0.637	1.85	±	1.01	0.226	0.04	±	1.12	0.934	−0.15	±	0.87	0.459
	LJ88	52	2.13	±	1.12	2.13	±	1.09	0.906	1.90	±	1.09	0.070	0.00	±	0.97		−0.23	±	0.83	
11. Increased passage of stools	Placebo	55	1.67	±	0.88	1.49	±	0.77	0.080	1.62	±	0.91	0.590	−0.18	±	0.75	0.436	−0.05	±	0.91	0.339
	LJ88	52	1.58	±	0.89	1.46	±	0.78	0.351	1.38	±	0.87	0.151	−0.12	±	0.83		−0.19	±	0.91	
12. Loose stools	Placebo	55	1.73	±	0.93	1.67	±	0.88	0.665	1.73	±	0.95	0.944	−0.05	±	0.80	0.663	0.00	±	1.00	0.099
	LJ88	52	1.71	±	0.91	1.69	±	1.02	0.983	1.38	±	0.84	0.006	−0.02	±	0.80		−0.33	±	0.81	
13. Hard stools	Placebo	55	1.96	±	1.22	1.87	±	1.02	0.649	1.75	±	0.99	0.162	−0.09	±	1.09	0.883	−0.22	±	1.05	0.970
	LJ88	52	1.87	±	0.84	1.87	±	1.16	0.927	1.79	±	1.16	0.321	0.00	±	0.93		−0.08	±	0.90	
14. Urgent need for defecation	Placebo	55	2.00	±	1.00	1.82	±	1.06	0.154	1.78	±	1.10	0.067	−0.18	±	1.00	0.604	−0.22	±	0.96	0.488
	LJ88	52	1.92	±	1.13	1.79	±	1.09	0.234	1.77	±	1.04	0.198	−0.13	±	0.91		−0.15	±	0.83	
15. Feeling of incomplete evacuation	Placebo	55	2.60	±	1.37	2.44	±	1.33	0.404	2.45	±	1.21	0.277	−0.16	±	1.13	0.720	−0.15	±	0.95	0.603
	LJ88	52	2.48	±	1.26	2.44	±	1.19	0.719	2.17	±	1.12	0.043	−0.04	±	1.14		−0.31	±	1.06	
Total score	Placebo	55	31.95	±	9.18	29.87	±	8.41	0.029	28.33	±	9.33	0.001	−2.07	±	6.68	0.699	−3.62	±	7.52	0.422
	LJ88	52	32.17	±	8.60	30.63	±	9.62	0.149	27.58	±	8.31	0.000	−1.54	±	6.64		−4.60	±	6.93	

^(1)^ Wilcoxon signed-rank test (vs. 0 W) with Bonferroni correction. ^(2)^ Wilcoxon rank sum test (between groups).

**Table 5:** GSRS relief rate (per-protocol set).

Items	Group	n	Relief Rate ^(1)^					
			0 W to 3 W			0 W to 6 W		
			Not Alleviated	Alleviated	*p* Value ^(2)^	Not Alleviated	Alleviated	*p* Value ^(2)^
1. Abdominal pain	Placebo	55	41	14	1.000	40	15	0.530
	LJ88	52	38	14		34	18	
2. Heartburn	Placebo	55	33	22	1.000	28	27	0.563
	LJ88	52	32	20		23	29	
3. Acid regurgitation	Placebo	55	40	15	1.000	32	23	0.844
	LJ88	52	37	15		32	20	
4. Sucking sensations in the epigastrium	Placebo	55	40	15	0.673	35	20	0.558
	LJ88	52	35	17		30	22	
5. Nausea and vomiting	Placebo	55	40	15	0.162	39	16	0.266
	LJ88	52	44	8		42	10	
6. Borborygmus	Placebo	55	40	15	0.367	37	18	0.839
	LJ88	52	42	10		36	16	
7. Abdominal distension	Placebo	55	33	22	1.000	32	23	0.336
	LJ88	52	31	21		25	27	
8. Eructation	Placebo	55	38	17	0.836	35	20	0.436
	LJ88	52	37	15		29	23	
9. Increased flatus	Placebo	55	40	15	1.000	34	21	0.697
	LJ88	52	38	14		30	22	
10 Decreased passage of stools	Placebo	55	43	12	1.000	38	17	0.837
	LJ88	52	41	11		34	18	
11. Increased passage of stools	Placebo	55	41	14	0.824	43	12	0.653
	LJ88	52	40	12		38	14	
12. Loose stools	Placebo	55	43	12	0.813	42	13	0.390
	LJ88	52	42	10		35	17	
13. Hard stools	Placebo	55	43	12	0.820	38	17	1.000
	LJ88	52	39	13		36	16	
14. Urgent need for defecation	Placebo	55	35	20	0.217	33	22	0.219
	LJ88	52	39	13		38	14	
15. Feeling of incomplete evacuation	Placebo	55	37	18	0.839	37	18	0.690
	LJ88	52	36	16		33	19	
Total score	Placebo	55	24	31	0.700	19	36	0.300
	LJ88	52	25	27		13	39	

^(1)^ Relief rate is the number of participants whose symptoms were alleviated or not at 3 W or 6 W compared to 0 W based on the changes in GSRS scores. ^(2)^ Fisher’s exact test.

**Table 6:** Questionnaire on the stomach condition (per-protocol set).

Group	n	Stomach State Questionnaire ^(1)^ (Score)								Stomach State Questionnaire ^(1)^ (Relief Rate)					
		3 W				6 W				3 W				6 W	
		Mean	±	SD	*p* Value ^(2)^	Mean	±	SD	*p* Value ^(2)^	Not Alleviated	Alleviated	*p* Value ^(3)^	Not Alleviated	Alleviated	*p* Value ^(3)^
Placebo	55	2.44	±	0.54	0.816	2.38	±	0.68	0.939	25	30	0.846	25	30	1.000
LJ88	52	2.42	±	0.57		2.40	±	0.63		22	30		23	29	

^(1)^ Questionnaire with five grades (1: very improved; 2: slightly improved; 3: no change; 4: slightly worsened; 5: very worsened; Alleviated: 1 and 2). ^(2)^ Wilcoxon rank sum test (between groups). ^(3)^ Fisher’s exact test.

**Table 7:** Change in serum gastrin concentration (per-protocol set).

Group	Gastrin Concentration (pmoles/L)									Change in Gastrin Concentration n				
	n	0 W			n	6 W				n	0–6 W			
		Mean	±	SD		Mean	±	SD	*p* Value ^(1)^		Mean	±	SD	*p* Value ^(2)^
Placebo	55	20.3	±	4.9	55	21.4	±	4.3	0.099	55	1.2	±	5.2	0.311
LJ88	52	27.7	±	34.3	52	26.4	±	27.1	0.824	52	−1.2	±	9.3	

^(1)^ Wilcoxon signed-rank test. ^(2)^ Wilcoxon rank sum test.

**Table 8:** Change in POMS-2 score (per-protocol set).

Items	Group	n	POMS-2 Scores							Change in POMS-2 Scores			
			0 W			6 W				0–6 W			
			Mean	±	SD	Mean	±	SD	*p* Value ^(1)^	Mean	±	SD	*p* Value ^(2)^
Anger-hostility	Placebo	55	47.64	±	9.08	47.13	±	8.10	0.492	−0.51	±	5.45	0.906
	LJ88	52	46.35	±	8.07	45.96	±	8.83	0.614	−0.38	±	5.47	
Confusion-bewilderment	Placebo	55	49.36	±	9.33	49.38	±	9.80	0.983	0.02	±	6.14	0.349
	LJ88	52	47.77	±	8.51	46.71	±	7.35	0.184	−1.06	±	5.67	
Depression-dejection	Placebo	55	48.67	±	8.49	49.20	±	9.40	0.583	0.53	±	7.07	0.260
	LJ88	52	46.04	±	6.53	45.29	±	4.79	0.218	−0.75	±	4.34	
Fatigue-inertia	Placebo	55	47.69	±	8.99	47.84	±	10.26	0.870	0.15	±	6.54	0.663
	LJ88	52	46.42	±	8.41	46.06	±	8.17	0.633	−0.37	±	5.48	
Tension-anxiety	Placebo	55	49.27	±	8.75	49.11	±	10.14	0.861	−0.16	±	6.87	0.577
	LJ88	52	46.56	±	8.82	47.04	±	8.94	0.484	0.48	±	4.91	
Vigor-activity	Placebo	55	53.04	±	10.25	51.95	±	10.08	0.166	−1.09	±	5.77	0.097
	LJ88	52	53.48	±	9.83	54.56	±	11.55	0.309	1.08	±	7.56	
Friendliness	Placebo	55	55.53	±	10.01	53.33	±	10.19	0.007	−2.20	±	5.86	0.116
	LJ88	52	53.75	±	9.13	53.56	±	10.70	0.848	−0.19	±	7.21	
Total mood disturbance	Placebo	55	47.80	±	8.29	48.02	±	9.64	0.760	0.22	±	5.27	0.370
	LJ88	52	45.71	±	7.60	45.08	±	6.36	0.311	−0.63	±	4.48	

^(1)^ Student’s *t*-test (paired). ^(2)^ Student’s *t*-test (unpaired).

**Table 9:** Change in SF-36v2 scores (per-protocol set).

Items	Group	n	SF-36v2 Scores							Change in SF-36v2 Scores			
			0 W			6 W				0–6 W			
			Mean	±	SD	Mean	±	SD	*p* Value ^(1)^	Mean	±	SD	*p* Value ^(2)^
Physical functioning	Placebo	55	54.44	±	4.01	54.38	±	4.06	0.909	−0.05	±	3.42	0.225
	LJ88	52	53.76	±	4.63	54.49	±	3.93	0.106	0.73	±	3.21	
Role physical	Placebo	55	54.08	±	5.24	53.03	±	6.19	0.098	−1.05	±	4.62	0.057
	LJ88	52	53.98	±	5.92	54.59	±	4.38	0.309	0.61	±	4.29	
Bodily Pain	Placebo	55	53.39	±	8.16	53.27	±	7.61	0.888	−0.12	±	6.38	0.163
	LJ88	52	51.77	±	8.91	53.67	±	8.01	0.109	1.89	±	8.38	
General health	Placebo	55	53.85	±	8.01	54.93	±	8.47	0.249	1.07	±	6.83	0.628
	LJ88	52	55.68	±	8.26	57.36	±	7.81	0.050	1.68	±	6.02	
Vitality	Placebo	55	50.61	±	8.37	51.33	±	8.75	0.389	0.72	±	6.17	0.784
	LJ88	52	49.84	±	9.17	50.90	±	8.19	0.239	1.06	±	6.39	
Social functioning	Placebo	55	53.40	±	6.77	54.22	±	6.46	0.433	0.82	±	7.69	0.350
	LJ88	52	53.58	±	6.80	52.72	±	10.12	0.563	−0.86	±	10.64	
Role emotional	Placebo	55	52.41	±	6.98	51.93	±	7.90	0.547	−0.47	±	5.77	0.799
	LJ88	52	53.58	±	7.02	53.44	±	4.74	0.889	−0.14	±	7.40	
Mental health	Placebo	55	53.02	±	6.80	52.88	±	8.05	0.859	−0.14	±	5.69	0.424
	LJ88	52	54.24	±	7.26	55.02	±	6.43	0.363	0.78	±	6.13	
Physical Component Summary (three components)	Placebo	55	54.17	±	5.65	54.05	±	5.49	0.850	−0.11	±	4.32	0.050
	LJ88	52	53.19	±	6.32	55.10	±	6.88	0.029	1.92	±	6.15	
Mental Component Summary (three components)	Placebo	55	51.25	±	7.11	52.31	±	7.93	0.169	1.06	±	5.62	0.808
	LJ88	52	51.82	±	8.26	53.15	±	7.35	0.115	1.33	±	5.98	
Role-social Component Summary (three components)	Placebo	55	52.09	±	5.97	51.26	±	7.58	0.318	−0.84	±	6.17	0.622
	LJ88	52	52.77	±	7.45	51.24	±	7.04	0.184	−1.53	±	8.19	
Physical Component Summary Universal (two components)	Placebo	55	54.22	±	5.07	54.19	±	4.90	0.958	−0.03	±	3.56	0.049
	LJ88	52	53.32	±	5.65	54.76	±	5.34	0.014	1.44	±	4.07	
Mental Component Summary Universal (two components)	Placebo	55	51.65	±	6.93	51.90	±	7.85	0.733	0.25	±	5.40	0.763
	LJ88	52	52.91	±	7.50	52.78	±	6.21	0.900	−0.13	±	7.48	
Physical Component Summary Japanese (two components)	Placebo	55	54.53	±	5.74	53.75	±	5.94	0.147	−0.77	±	3.90	0.182
	LJ88	52	54.18	±	5.62	54.56	±	4.60	0.586	0.37	±	4.88	
Mental Component Summary Japanese (two components)	Placebo	55	51.64	±	7.00	52.53	±	7.71	0.230	0.90	±	5.47	0.925
	LJ88	52	52.23	±	7.94	53.23	±	7.14	0.259	1.00	±	6.34	

^(1)^ Student’s *t*-test (paired). ^(2)^ Student’s *t*-test (unpaired).

## Data Availability

The data used in this manuscript are not publicly available because of participants’ privacy concerns, but are available on reasonable request.

## Supplementary Materials

The following supporting information can be downloaded at: https://www.mdpi.com/article/10.3390/nu16081230/s1, Table S1: Baseline characteristics of participants (Blood test and urinalysis) (full analysis set); Table S2: Change in body weight, BMI, blood pressures, and pulse rate (full analysis set); Table S3: Blood biochemical test values (full analysis set); Table S4: Change in blood cellular tests results (full analysis set); Table S5: Change in urinalysis results (full analysis set).
