# Supplementary material for: Beneficial Effect of Heat-Killed Lactic Acid Bacterium Lactobacillus johnsonii No. 1088 on Temporal Gastroesophageal Reflux-Related Symptoms in Healthy Volunteers: A Randomized, Placebo-Controlled, Double-Blind, Parallel-Group Study
Source: Nutrients. 2024 Apr 20;16(8):1230. doi: 10.3390/nu16081230 (PMC11054138; doi:10.3390/nu16081230)
Supplement: Supplementary file 1 [file nutrients-16-01230-s001.zip › Table S1.pdf]

Table S1. Baseline characteristics of participants (Blood test and urinalysis)(full analysis set)

Items		Placebo group (n = 60)	LJ88 group (n = 60)	p-value	(method)[1]	All participants (n = 120)
White blood cell count (/μL)		5283 ± 1315	5697 ± 1561	0.119		5490 ± 1452
Number of red blood cells ($\times 10^4$/μL)		450.8 ± 53.2	456.4 ± 46.5	0.543		453.6 ± 49.9
Hemoglobin (g/dL)		13.7 ± 1.5	13.7 ± 1.1	0.939		13.7 ± 1.3
Hematocrit (%)		43.0 ± 4.0	43.5 ± 3.4	0.476		43.3 ± 3.7
Platelet count ($\times 10^4$/μL)		25.9 ± 6.2	25.8 ± 5.6	0.930		25.8 ± 5.9
Mean Corpuscular Volume (MCV) (fL)		95.8 ± 5.3	95.7 ± 4.7	0.884		95.8 ± 5.0
Mean Corpuscular Hemoglobin(MCH) (pg)		30.4 ± 1.8	30.1 ± 1.6	0.290		30.3 ± 1.7
Mean Corpuscular Hemoglobin Consentration (MCHC) (%)		31.8 ± 0.9	31.5 ± 1.0	0.119		31.6 ± 1.0
Neutrophils/white blood cell image (%)		56.5 ± 6.9	57.1 ± 8.1	0.693		56.8 ± 7.5
Lymphocytes/white blood cell image (%)		33.6 ± 6.2	33.6 ± 7.5	0.976		33.6 ± 6.8
Monocytes/white blood cell image (%)		5.9 ± 1.6	5.6 ± 1.3	0.217		5.7 ± 1.5
Eosinophils/white blood cell image (%)		3.1 ± 2.1	3.0 ± 2.0	0.696		3.0 ± 2.1
Basophils/white blood cell image (%)		0.9 ± 0.4	0.8 ± 0.4	0.718		0.8 ± 0.4
Total protein (g/dL)		7.0 ± 0.4	7.0 ± 0.4	0.796		7.0 ± 0.4
Albumin (g/dL)		4.4 ± 0.3	4.4 ± 0.3	0.793		4.4 ± 0.3
Aspartate aminotransferase（AST) (U/L)		20.2 ± 4.4	20.2 ± 6.5	0.935		20.2 ± 5.5
Alanine aminotransferas (ALT) (U/L)		17.1 ± 8.2	16.9 ± 7.8	0.882	(Student's t-test)	17.0 ± 8.0
Lactate dehydorogenase (IFCC) (U/L)		173.7 ± 24.4	172.1 ± 24.2	0.711		172.9 ± 24.2
Total bilibin (mg/dL)		0.9 ± 0.3	0.8 ± 0.3	0.367		0.9 ± 0.3
Alkaline phosphatase (IFCC) (U/L)		60.9 ± 17.4	64.2 ± 17.1	0.295		62.5 ± 17.3
γ-Glutamyltransferas (U/L)		24.9 ± 15.2	21.8 ± 14.9	0.270		23.4 ± 15.1
Urea nitrogen (UN) (mg/dL)		13.1 ± 4.1	13.1 ± 3.5	0.960		13.1 ± 3.8
Creatinine (mg/dL)		0.77 ± 0.17	0.75 ± 0.14	0.584		0.76 ± 0.16
Uric acid (UA) (mg/dL)		5.1 ± 1.3	5.3 ± 1.3	0.558		5.2 ± 1.3
Sodium (Na) (mEq/L)		140.2 ± 1.8	140.3 ± 1.6	0.831		140.2 ± 1.7
Chlorine (Cl) (mEq/L)		103.2 ± 1.7	103.3 ± 1.7	0.915		103.2 ± 1.7
Potassium (K) mEq/L)		4.1 ± 0.3	4.1 ± 0.3	0.491		4.1 ± 0.3
Calcium (Ca) (mg/dL)		9.3 ± 0.3	9.4 ± 0.3	0.574		9.4 ± 0.3
Total cholesterol (mg/dL)		195.3 ± 26.3	199.8 ± 32.7	0.411		197.5 ± 29.6
LDL-cholesterol (mg/dL)		113.2 ± 27.2	115.7 ± 27.0	0.612		114.5 ± 27.0
HDL-cholesterol (mg/dL)		67.8 ± 16.3	70.8 ± 20.2	0.370		69.3 ± 18.3
TG (neutral fat) (mg/dL)		81.3 ± 42.8	81.2 ± 42.9	0.981		81.2 ± 42.7
Glucose (mg/dL)		84.9 ± 6.8	85.3 ± 7.9	0.766		85.1 ± 7.3
HbA1c(NGSP) (%)		5.3 ± 0.3	5.4 ± 0.2	0.096		5.4 ± 0.2
urine pH		6.18 ± 0.51	6.24 ± 0.65	0.535		6.21 ± 0.59
urine specific gravity		1.018 ± 0.009	1.019 ± 0.009	0.561		1.019 ± 0.009
Menstration	Before menopause	20	19			39
	Merginal (2 times/mt)	0	1	1.000		1
	After menopause	13	14		(Fisher's exact test)	27
Urine protein	-	57	57			114
	+/-	2	3	1.000		5
	3+	1	0			1
Urine glucose	-	60	60	-		120
Urine urobilinogen	+/-	60	60	-	-	120
Urine bilirubin	-	60	60	-		120
Urine keton body	-	60	60	-		120
Urinary occult blood reaction	-	55	60			115
	+/-	1	0			1
	+	2	0	0.057	(Fisher's exact test)	2
	2+	1	0			1
	3+	1	0			1

Mean ± SD or number of participants are depicted.

[1]Methods for statistical analyses
