# Supplementary material for: Beneficial Effect of Heat-Killed Lactic Acid Bacterium Lactobacillus johnsonii No. 1088 on Temporal Gastroesophageal Reflux-Related Symptoms in Healthy Volunteers: A Randomized, Placebo-Controlled, Double-Blind, Parallel-Group Study
Source: Nutrients. 2024 Apr 20;16(8):1230. doi: 10.3390/nu16081230 (PMC11054138; doi:10.3390/nu16081230)
Supplement: Supplementary file 1 [file nutrients-16-01230-s001.zip › Table S2.pdf]

Table S2. Change in body weight, BMI, blood pressures, and pulse rate (full analysis set)

Parameter	Group	n	0W			n	3W				n	6W			
			mean	±	SD		mean	±	SD	*p* value[1)]		mean	±	SD	*p* value[1)]
Body weight (kg)	Placebo	60	60.8	±	11.7	60	61.1	±	11.6	0.002	60	61.0	±	11.6	0.100
	LJ88	60	60.4	±	11.5	60	60.5	±	11.3	0.933	59	60.3	±	11.3	0.580
BMI	Placebo	60	22.3	±	3.2	60	22.4	±	3.1	0.001	60	22.4	±	3.1	0.063
	LJ88	60	22.2	±	3.2	60	22.2	±	3.2	0.922	59	22.1	±	3.2	0.515
Systolic blood pressure (mmHg)	Placebo	60	125.4	±	15.8	60	122.3	±	14.3	0.067	60	125.7	±	14.6	0.984
	LJ88	60	120.0	±	14.5	60	118.9	±	13.0	0.610	59	122.0	±	14.0	0.234
Diastolic blood pressure (mmHg)	Placebo	60	79.9	±	12.5	60	76.0	±	13.1	0.000	60	80.6	±	12.8	0.706
	LJ88	60	76.7	±	11.2	60	73.2	±	9.6	0.002	59	76.7	±	9.3	0.999
Pulse rate (bpm)	Placebo	60	75.3	±	9.5	60	74.2	±	9.6	0.555	60	76.2	±	9.0	0.650
	LJ88	60	76.2	±	11.2	60	74.3	±	10.1	0.218	59	77.0	±	12.2	0.690

[1)]Dunnet test (vs. 0W).
