# Supplementary material for: Beneficial Effect of Heat-Killed Lactic Acid Bacterium Lactobacillus johnsonii No. 1088 on Temporal Gastroesophageal Reflux-Related Symptoms in Healthy Volunteers: A Randomized, Placebo-Controlled, Double-Blind, Parallel-Group Study
Source: Nutrients. 2024 Apr 20;16(8):1230. doi: 10.3390/nu16081230 (PMC11054138; doi:10.3390/nu16081230)
Supplement: Supplementary file 1 [file nutrients-16-01230-s001.zip › Table S3.pdf]

Table S3. Blood biochemical test values (full analysis set)

Items	Group	n	At screening			n	6W			
			mean	±	SD		mean	±	SD	*p* value[1)]
Aspartate aminotransferase（AST) (U/L)	Placebo	60	20.2	±	4.4	60	19.9	±	4.0	0.704
	LJ88	60	20.2	±	6.5	59	20.8	±	6.7	0.467
Alanine aminotransferas (ALT) (U/L)	Placebo	60	17.1	±	8.2	60	18.0	±	8.2	0.311
	LJ88	60	16.9	±	7.8	59	18.7	±	9.3	0.020
Lactate dehydorogenase (IFCC) (U/L)	Placebo	60	173.7	±	24.4	60	187.6	±	27.3	0.000
	LJ88	60	172.1	±	24.2	59	182.9	±	28.7	0.000
Total bilibin (mg/dL)	Placebo	60	0.9	±	0.3	60	0.8	±	0.2	0.045
	LJ88	60	0.8	±	0.3	59	0.8	±	0.3	0.088
Alkaline phosphatase (IFCC) (U/L)	Placebo	60	60.9	±	17.4	60	64.7	±	17.7	0.000
	LJ88	60	64.2	±	17.1	59	68.6	±	20.0	0.000
γ-Glutamyltransferas (U/L)	Placebo	60	24.9	±	15.2	60	25.0	±	15.3	0.921
	LJ88	60	21.8	±	14.9	59	21.8	±	14.4	0.597
Glucose (mg/dL)	Placebo	60	84.9	±	6.8	60	85.6	±	8.3	0.416
	LJ88	60	85.3	±	7.9	59	82.7	±	8.1	0.036
HbA1c(NGSP) (%)	Placebo	60	5.3	±	0.3	60	5.4	±	0.3	0.000
	LJ88	60	5.4	±	0.2	59	5.5	±	0.2	0.000
Total cholesterol (mg/dL)	Placebo	60	195.3	±	26.3	60	207.4	±	30.3	0.000
	LJ88	60	199.8	±	32.7	59	208.1	±	33.6	0.002
LDL-cholesterol (mg/dL)	Placebo	60	113.2	±	27.2	60	120.1	±	29.0	0.003
	LJ88	60	115.7	±	27.0	59	119.2	±	27.8	0.076
HDL-cholesterol (mg/dL)	Placebo	60	67.8	±	16.3	60	70.3	±	19.2	0.015
	LJ88	60	70.8	±	20.2	59	71.8	±	19.9	0.496
TG (neutral fat) (mg/dL)	Placebo	60	81.3	±	42.8	60	86.4	±	57.8	0.355
	LJ88	60	81.2	±	42.9	59	90.7	±	50.1	0.090
Total protein (g/dL)	Placebo	60	7.0	±	0.4	60	7.3	±	0.4	0.000
	LJ88	60	7.0	±	0.4	59	7.2	±	0.3	0.000
Albumin (g/dL)	Placebo	60	4.4	±	0.3	60	4.4	±	0.2	0.365
	LJ88	60	4.4	±	0.3	59	4.4	±	0.3	0.348
Urea nitrogen (UN) (mg/dL)	Placebo	60	13.1	±	4.1	60	12.5	±	3.4	0.106
	LJ88	60	13.1	±	3.5	59	13.0	±	3.5	0.589
Creatinine (mg/dL)	Placebo	60	0.8	±	0.2	60	0.7	±	0.2	0.000
	LJ88	60	0.8	±	0.1	59	0.7	±	0.2	0.005
Uric acid (UA) (mg/dL)	Placebo	60	5.1	±	1.3	60	5.0	±	1.2	0.186
	LJ88	60	5.3	±	1.3	59	5.2	±	1.3	0.366
Sodium (Na) (mEq/L)	Placebo	60	140.2	±	1.8	60	140.5	±	1.9	0.254
	LJ88	60	140.3	±	1.6	59	141.0	±	1.7	0.007
Chlorine (Cl) (mEq/L)	Placebo	60	103.2	±	1.7	60	104.0	±	1.8	0.004
	LJ88	60	103.3	±	1.7	59	104.1	±	1.7	0.001
Potassium (K) (mEq/L)	Placebo	60	4.1	±	0.3	60	4.2	±	0.3	0.002
	LJ88	60	4.1	±	0.3	59	4.2	±	0.3	0.167
Calcium (Ca) (mg/dL)	Placebo	60	9.3	±	0.3	60	9.4	±	0.3	0.103
	LJ88	60	9.4	±	0.3	59	9.4	±	0.3	0.825

[1)] Studen's t-teat (paired)
