# Supplementary material for: Beneficial Effect of Heat-Killed Lactic Acid Bacterium Lactobacillus johnsonii No. 1088 on Temporal Gastroesophageal Reflux-Related Symptoms in Healthy Volunteers: A Randomized, Placebo-Controlled, Double-Blind, Parallel-Group Study
Source: Nutrients. 2024 Apr 20;16(8):1230. doi: 10.3390/nu16081230 (PMC11054138; doi:10.3390/nu16081230)
Supplement: Supplementary file 1 [file nutrients-16-01230-s001.zip › Table S4.pdf]

Table S4. Change in blood cellular tests results (full analysis set)

Items	Group	n	At screening			n	6W			
			mean	±	SD		mean	±	SD	*p* value[1)]
White blood cell count (/μL)	Placebo	60	5283	±	1315	60	5510	±	1434	0.141
	LJ88	60	5697	±	1561	59	5592	±	1448	0.735
Number of red blood cells (×10⁴/μL)	Placebo	60	450.8	±	53.2	60	469.0	±	49.6	0.000
	LJ88	60	456.4	±	46.5	59	467.1	±	47.1	0.003
Hemoglobin (g/dL)	Placebo	60	13.7	±	1.5	60	14.2	±	1.4	0.000
	LJ88	60	13.7	±	1.1	59	14.0	±	1.2	0.002
Hematocrit (%)	Placebo	60	43.0	±	4.0	60	44.2	±	3.9	0.000
	LJ88	60	43.5	±	3.4	59	43.6	±	3.6	0.907
Platelet count (×10⁴/μL)	Placebo	60	25.9	±	6.2	60	27.1	±	6.1	0.004
	LJ88	60	25.8	±	5.6	59	27.4	±	7.9	0.000
Mean Corpuscular Volume (MCV) (fL)	Placebo	60	95.8	±	5.3	60	94.4	±	4.6	0.000
	LJ88	60	95.7	±	4.7	59	93.5	±	4.4	0.000
Mean Corpuscular Hemoglobin(MCH) (pg)	Placebo	60	30.4	±	1.8	60	30.4	±	1.7	0.594
	LJ88	60	30.1	±	1.6	59	30.1	±	1.6	0.789
Mean Corpuscular Hemoglobin Consentration (MCHC) (%)	Placebo	60	31.8	±	0.9	60	32.2	±	0.9	0.001
	LJ88	60	31.5	±	1.0	59	32.2	±	0.9	0.000
Neutrophils/white blood cell image (%)	Placebo	60	56.5	±	6.9	60	58.2	±	8.6	0.118
	LJ88	60	57.1	±	8.1	59	57.0	±	7.5	0.891
Lymphocytes/white blood cell image (%)	Placebo	60	33.6	±	6.2	60	32.4	±	7.6	0.174
	LJ88	60	33.6	±	7.5	59	33.4	±	7.0	0.698
Monocytes/white blood cell image (%)	Placebo	60	5.9	±	1.6	60	5.7	±	1.7	0.274
	LJ88	60	5.6	±	1.3	59	5.8	±	1.5	0.047
Eosinophils/white blood cell image (%)	Placebo	60	3.1	±	2.1	60	3.0	±	2.1	0.448
	LJ88	60	3.0	±	2.0	59	3.0	±	1.9	0.802
Basophils/white blood cell image (%)	Placebo	60	0.9	±	0.4	60	0.8	±	0.3	0.339
	LJ88	60	0.8	±	0.4	59	0.8	±	0.4	0.488

[1)]Studen's t-teat (paired)
