# Supplementary material for: Beneficial Effect of Heat-Killed Lactic Acid Bacterium Lactobacillus johnsonii No. 1088 on Temporal Gastroesophageal Reflux-Related Symptoms in Healthy Volunteers: A Randomized, Placebo-Controlled, Double-Blind, Parallel-Group Study
Source: Nutrients. 2024 Apr 20;16(8):1230. doi: 10.3390/nu16081230 (PMC11054138; doi:10.3390/nu16081230)
Supplement: Supplementary file 1 [file nutrients-16-01230-s001.zip › Table S5.pdf]

**Table S5. Change in urinalysis results (full analysis set)**

| Items | Group | n | At screening | | | n | 6W | | | | |
|---|---|---|---|---|---|---|---|---|---|---|---|
| | | | mean | ± | SD | | mean | ± | SD | *p* value[1)] | (method) |
| Urine pH | Placebo | 60 | 6.18 | ± | 0.51 | 60 | 6.18 | ± | 0.54 | 0.922 | (Student's t-test) |
| | LJ88 | 60 | 6.24 | ± | 0.65 | 59 | 6.17 | ± | 0.51 | 0.533 | |
| Urine specific gravity | Placebo | 60 | 1.018 | ± | 0.009 | 60 | 1.017 | ± | 0.007 | 0.176 | |
| | LJ88 | 60 | 1.019 | ± | 0.009 | 59 | 1.019 | ± | 0.009 | 0.793 | |
| Urine protein (qualitative)* | Placebo | 60 | 0.10 | ± | 0.54 | 60 | 0.05 | ± | 0.29 | 0.680 | (Wilcoxon signed-rank test) |
| | LJ88 | 60 | 0.05 | ± | 0.22 | 59 | 0.05 | ± | 0.22 | 1.000 | |
| Urine glucose (qualitative)* | Placebo | 60 | 0.00 | ± | 0.00 | 60 | 0.00 | ± | 0.00 | 1.000 | |
| | LJ88 | 60 | 0.00 | ± | 0.00 | 59 | 0.00 | ± | 0.00 | 1.000 | |
| Urine urobilinogen (qualitative)* | Placebo | 60 | 0.00 | ± | 0.00 | 60 | 0.00 | ± | 0.00 | 1.000 | |
| | LJ88 | 60 | 0.00 | ± | 0.00 | 59 | 0.00 | ± | 0.00 | 1.000 | |
| Urine bilirubin (qualitative)* | Placebo | 60 | 0.00 | ± | 0.00 | 60 | 0.00 | ± | 0.00 | 1.000 | |
| | LJ88 | 60 | 0.00 | ± | 0.00 | 59 | 0.00 | ± | 0.00 | 1.000 | |
| Urine keton body* | Placebo | 60 | 0.00 | ± | 0.00 | 60 | 0.00 | ± | 0.00 | 1.000 | |
| | LJ88 | 60 | 0.00 | ± | 0.00 | 59 | 0.00 | ± | 0.00 | 1.000 | |
| Urinary occult blood reation* | Placebo | 60 | 0.20 | ± | 0.73 | 60 | 0.17 | ± | 0.69 | 0.596 | |
| | LJ88 | 60 | 0.00 | ± | 0.00 | 59 | 0.29 | ± | 0.87 | 0.016 | |

[1)]Qualitative data were scored and used for analyses.
